# An adult case of a retroperitoneal isolated enteric duplication cyst with the imaging changes over time

**DOI:** 10.1186/s40792-021-01337-x

**Published:** 2021-12-16

**Authors:** Mayu Inomata, Kengo Kai, Takuto Ikeda, Akiko Ichihara, Rie Masuda, Takumi Kiwaki, Hiroyuki Tanaka, Hiroaki Kataoka, Atsushi Nanashima

**Affiliations:** 1 Department of Surgery, University of Miyazaki Faculty of Medicine, 5200 Kihara, Kiyotake, Miyazaki, Miyazaki 889-1692 Japan; 2 Department of Radiology, University of Miyazaki Faculty of Medicine, 5200 Kihara, Kiyotake, Miyazaki, Miyazaki 889-1692 Japan; 3 Section of Oncopathology and Regenerative Biology, Department of Pathology, University of Miyazaki Faculty of Medicine, 5200 Kihara, Kiyotake, Miyazaki, Miyazaki 889-1692 Japan

**Keywords:** Isolated enteric duplication cyst, CT change of intra-cystic density, Laparoscopic surgery

## Abstract

**Background:**

Adult cases of retroperitoneal isolated enteric duplication cyst (IEDC) are rare, with only 17 case reports in the relevant literature. We herein present a case, which was characterized by changes in intra-cystic density on computed tomography (CT), which was safely resected by laparoscopic surgery.

**Case presentation:**

The patient was a 60-year-old male who received abdominal CT to investigate the cause of increased serum CA19-9 levels. CT revealed a unilocular cystic mass located in the lower right retroperitoneum. The size increased from 5 to 10 cm in three and a half years and the CT value decreased from 101 Hounsfield Units (HU) to 20 HU. We performed laparoscopic surgical resection, because the possibility that the enlargement of the lesion represented malignant transformation could not be denied. The large cystic mass firmly adhered to the appendix and its mesentery via the retroperitoneum, the appendix was resected en bloc with the cystic lesion. Microscopically, it had no communication with the appendix, and had an intestinal wall structure of muscularis mucosae and muscularis propria. The final pathological diagnosis was IEDC in the retroperitoneal space. There was no histological evidence of malignancy.

**Conclusion:**

When we encounter a retroperitoneal cystic lesion, we should consider the possibility of malignancy to determine the treatment strategy and perform a careful operation without breaking the cyst wall, irrespective of the preoperative diagnosis.

## Background

Enteric duplication cyst (EDC) is a congenital anomaly that can be found anywhere along the alimentary tract [1–3]. In almost all case, EDCs are attached or adjacent to a wall of the normal gastrointestinal tract; however, some cases lack anatomic association with the gastrointestinal tract and are called isolated EDCs (IEDCs) [4]. We experienced an adult case of IEDC. Interestingly, the density of the retroperitoneal IEDC changed from high to low on computed tomography (CT). Only 17 adult cases of retroperitoneal IEDC have been reported in the relevant literature. None of these reports described the imaging changes over time. We present a case in which the lesion was safely resected by laparoscopic surgery and summarize previous reports to grasp the clinical features of retroperitoneal IEDC.

## Case presentation

The patient was a 60-year-old male who regularly visited a general practitioner for type 2 diabetes, who performed routine examination to rule out malignant disease. As his serum carbohydrate antigen 19-9 (CA 19-9) level was slightly elevated, abdominal CT was performed, which revealed a unilocular cystic mass of 8.0 cm in diameter, located in the lower right retroperitoneum. He was, therefore, referred to our hospital for surgical treatment.

**Fig. 1:**
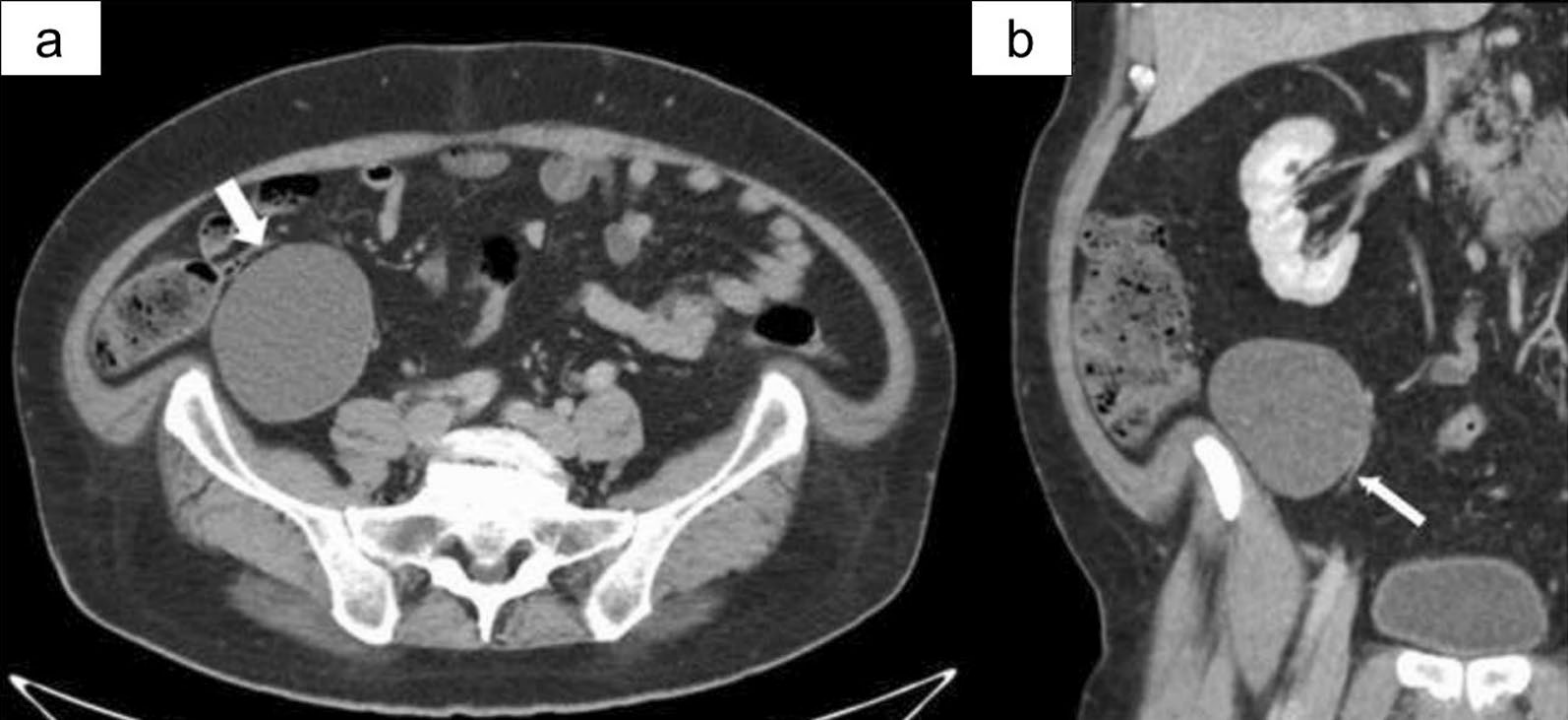
Preoperative computed tomography. Enhanced computed tomography showed a unilocular cystic mass (thick arrow) of 10 cm in diameter located below the right kidney in the retroperitoneum (**a**), which appeared to be close to the appendix on the ventral side and to the right gonadal vessel (thin arrow) on the medial side (**b**)

**Fig. 2:**
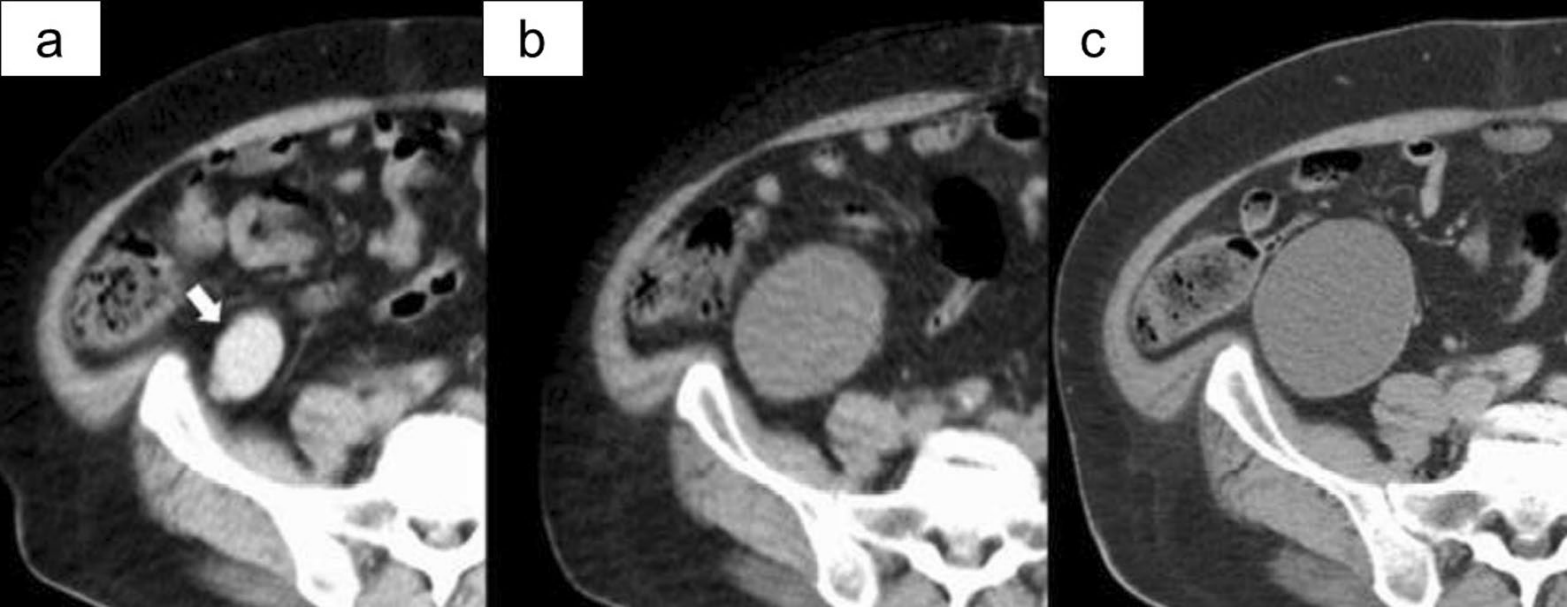
Computed tomography changed over time. Non-enhanced CT performed 3 years previously showed a 5-cm cystic mass with a uniform high density (arrow) (**a**), while non-enhanced CT performed 6 months previously showed a cystic mass had grown to 8 cm with a uniform low density (**b**). On the most recent CT scans, the concentration in the cyst was slightly reduced in comparison to these images (CT value: 20.3HU), and the diameter of the cyst had grown to 10 cm (**c**)

The patient had no relevant family history or past history of malignant disease in the abdomen or retroperitoneum. He was asymptomatic and in good health, with the exception of severe obesity with a body mass index of 40. An abdominal examination revealed no tenderness or palpable mass. Laboratory findings showed a high hemoglobin A1c level (7%; normal range 0–6%). The increased serum CA 19-9 levels, which was 43.3 U/mL in the previous hospital, had normalized to 16.9 U/mL at the time of the examination at our hospital. Ultrasonography identified a uniform hypoechoic cystic mass without septum and calcification in the retroperitoneum adjacent to the end of the ileum and appendix, which indicated a hyperechoic region with a debris-like appearance and no obvious nodular lesions or blood flow in the cyst. On enhanced CT, the retroperitoneal cystic mass appeared to be close to the appendix on the ventral side and to the right gonadal vessel on the medial side (Fig. 1a, b). Non-enhanced CT was regularly performed by his general practitioner who was managing him or diabetes to rule out a pancreatic lesion, and we also observed the changes of this cystic lesion over the course of three and a half years (Fig. 2a–c). The first image showed that the lesion was a 5 cm cystic mass with a uniform high density, with a CT value of 101 Hounsfield Units (HU). The second image, obtained 3 years later, showed that the cystic lesion had grown to 8 cm and that the internal density had decreased, as CT value of 28 HU. Eventually, the cyst diameter increased further to reach 10 cm and the CT value decreased slightly to 20 HU. Based on these clinical findings and the location, retroperitoneal lymphangioma, dermoid cyst and mucinous cystadenoma of appendix were listed as differential diagnoses. After we informed the patient that it would be difficult to make an accurate diagnosis of the retroperitoneal lesion and that malignant transformation could not be denied as a reason for the enlargement, we planned surgical resection rather than careful follow-up.

**Fig. 3:**
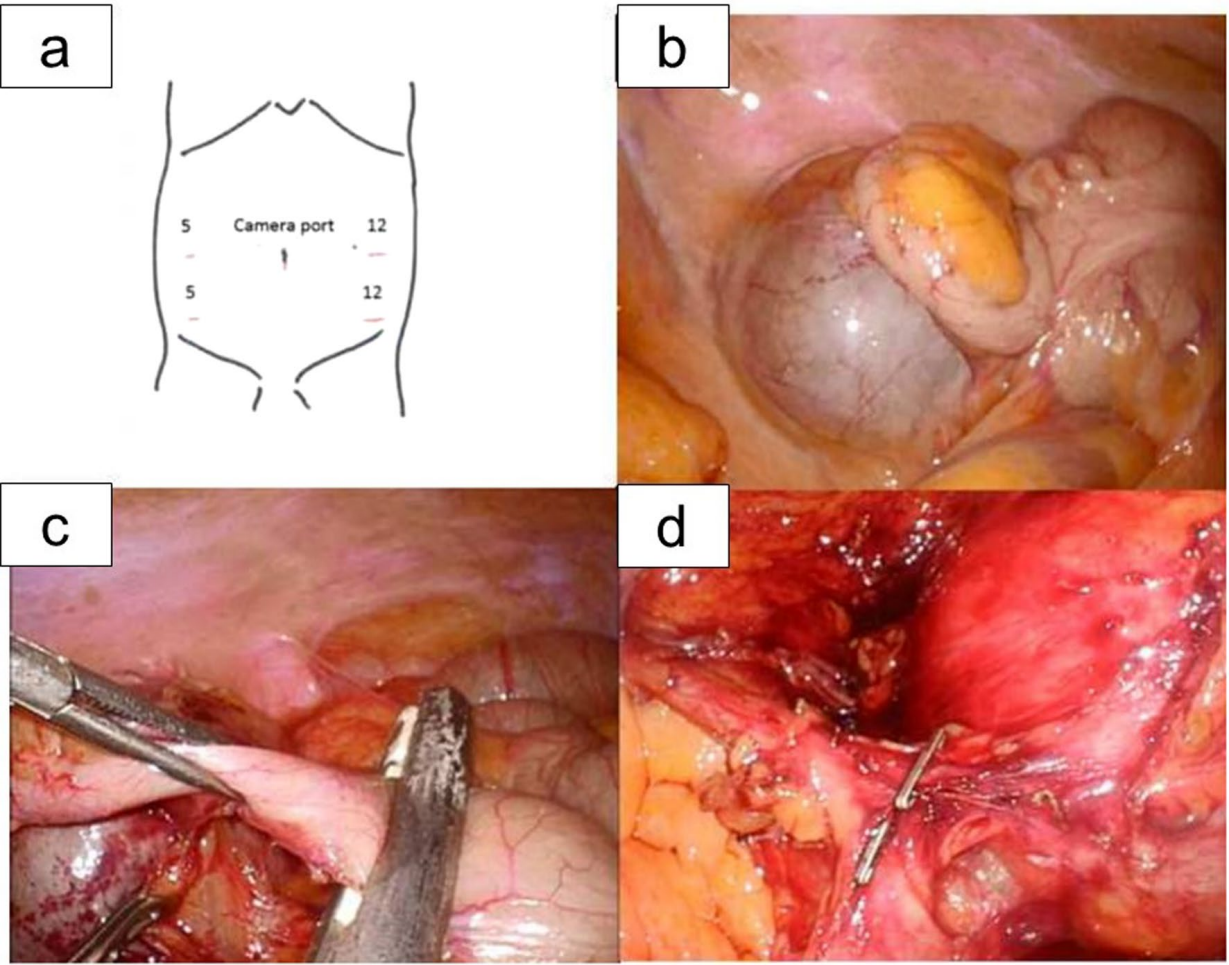
Laparoscopic resection of the isolated enteric duplication cyst. Laparoscopic resection was performed using 5 trocars (**a**). The large cystic mass was identified below the right kidney. It firmly adhered to the appendix and mesentery via the retroperitoneum (**b**). The appendix was dissected with a linear stapler and resected en bloc with the cystic lesion (**c**). Some feeding vessels from the right gonadal vessel were clipped and cut (**d**)

Laparoscopic surgery was performed under general anesthesia. The operative procedure began with the insertion of a 1.2 cm umbilical port using Hasson’s technique. Four additional ports were then inserted under direct visualization (Fig. 3a). The large cystic mass was identified in the retroperitoneal space below the right kidney and firmly adhered to the appendix and mesentery via the retroperitoneum (Fig. 3b). Due to strong adhesion, the appendix was dissected with a linear stapler and resected en bloc with the cystic lesion (Fig. 3c). After the lesion was mobilized from the retroperitoneal connective tissue, some feeding vessels from the right gonadal vessel were clipped and cut (Fig. 3d). The lesion was not associated with the colonic wall or ureter and could be completely dissected without injury to the surrounding organs. The lesion was placed in an entrapment endobag and removed from the abdomen via the umbilical port site. The total operative time was 157 min, and the estimated blood loss was small. Postoperatively, the patient made an uneventful recovery and was discharged home on postoperative day 4.

**Fig. 4:**
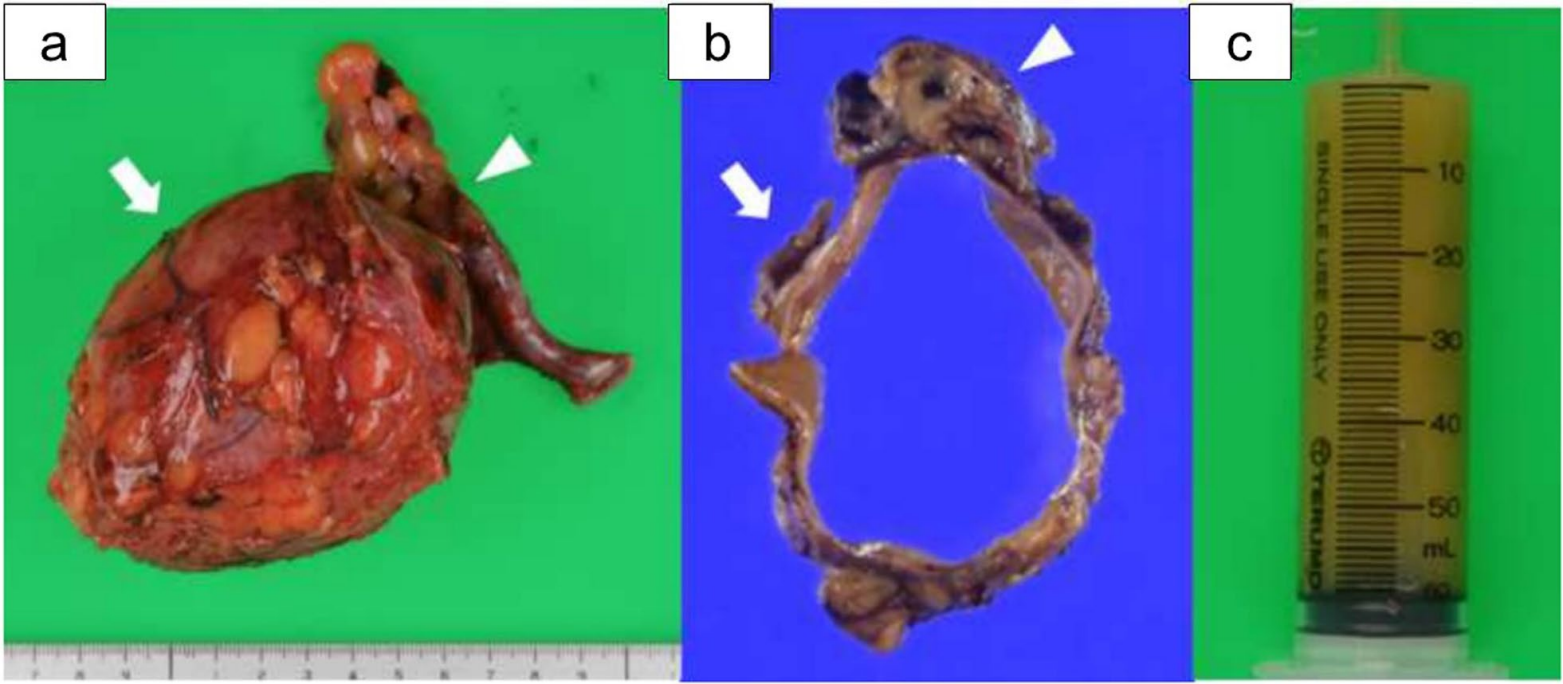
Resected specimen. The resected specimens consisted of a 10 × 8 × 8 cm unilocular cystic tumor with a smooth inner surface (arrow) and the appendix (arrow head) (**a**, **b**). The cystic lesion included a large amount of yellow, turbid serous fluid (**c**)

**Fig. 5:**
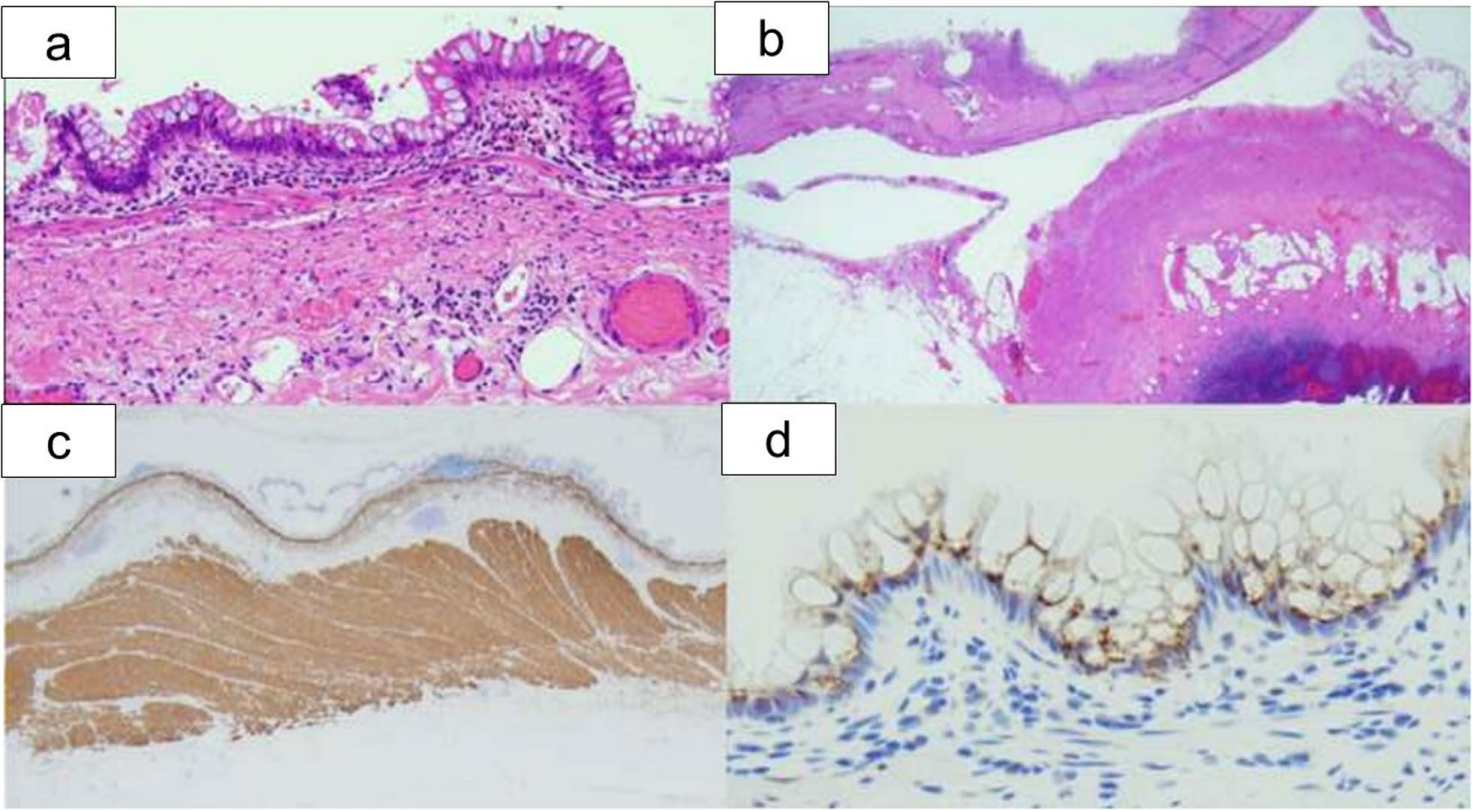
Histological staining. Hematoxylin and eosin staining demonstrated smooth muscular layers in the cystic wall, consistent with the muscularis mucosae, submucosa and muscularis propria of the gastrointestinal tract, without the communication to the appendix (**a**, **b**). Desmin staining confirmed the intestinal wall structure of the muscularis mucosae and muscularis propria (**c**). The lining epithelial cells were positive for Mucin 2, indicating an intestinal-type glandular mucosa (**d**)

The resected specimens consisted of a unilocular cystic tumor of 10 cm in maximum diameter with a smooth inner surface and the appendix (Fig. 4a, b). The cystic lesion included a large amount of yellow, turbid serous fluid (Fig. 4c). Microscopically, hematoxylin and eosin staining demonstrated the smooth muscular layers in the cystic wall, which was consistent with the muscularis mucosae, submucosa and muscularis propria of the gastrointestinal tract without communication with the appendix (Fig. 5a, b). Desmin staining confirmed the intestinal wall structure of the muscularis mucosae and the muscularis propria, which are needed to make histological diagnosis of EDC (Fig. 5c). The lining epithelial cells were positive for Mucin 2, indicating an intestinal-type glandular mucosa (Fig. 5d). The final pathological diagnosis was IEDC in the retroperitoneal space. There was no histological evidence of malignancy.

## Discussion

EDC is an uncommon congenital enteric malformation, the incidence rate of which is 1 in every 10,000 live births. Although most of patients with EDC are diagnosed in infancy or childhood, they can sometimes be seen in older patients [1, 2]. EDCs can occur in any portion of the alimentary tract; the most common location is the small bowel (jejuno-ileal: 47%), followed in order by the large bowel (20%), esophagus (17%), stomach (8%), and duodenum (5%). They tend to be located on the mesenteric side of the gastrointestinal tract and share its blood supply [5].

In 1961, Mellish and Koop reported that “enteric duplications” are defined histopathologically as spherical or tubular structures that possess a mucosal lining characteristic of 1 or more portions of the alimentary tract supported by muscular and serosal layers [6]. In our case, the EDC did not communicate with any gastrointestinal tract in the retroperitoneum. Such non-communicating, isolated EDCs have a wall with gastrointestinal epithelium and a well-developed coating of smooth muscle, similar to that seen in regular EDC, but without an anatomical association with the alimentary tract. This type of tumor has been reported in various locations, including the tongue [7], pleural space [8], liver [9], pancreas [10], biliary tree [11] and retroperitoneum (as is described in the presented case). IEDC in adults is very rare and the consensus on the diagnosis, including the characteristic feature of malignancy, has not been established.

To understand the clinical features of the disease, we searched the PubMed database cases of retroperitoneal IEDC. We identified 18 adult cases reports, including our case, between 1990 and 2020 and summarized these cases with the detailed clinical course (Table 1) [12–27]. The ages of the adult patients, including the present case, varied widely, with a median age of 39.9 years (range 19–75 years), and there was a female predominance, with women outnumbering men by a ratio of approximately 2:1. Surgical resection was performed in all reported cases, and laparoscopic surgery was reported to be associated with safe outcomes in recent cases [19, 22, 23, 25–27]. Since no patients have been accurately diagnosed preoperatively (including the present case), surgery was performed under diagnoses of cystic lesion of the retroperitoneal and pelvic organs, lymphangioma, teratoma, and infectious disease (e.g., retroperitoneal abscess). The reasons for surgical resection were not only symptoms, which included abdominal pain and palpable mass in many cases, but also because the possibility of malignant disease could not be ruled out in asymptomatic cases [15, 18, 23, 24]. In the present case, tumor enlargement was the only sign suggesting malignant potential. In fact, it seems difficult to preoperatively diagnose an IEDC as benign or malignant, because malignancy was not suspected before surgery in 5 of the 6 reported cases. Thus, to understand the characteristics of this tumor, we compare the clinical features of the reported malignant (*n* = 6) and non-malignant (*n* = 12) cases in Table 2, to evaluate factors that are useful for identifying retroperitoneal IEDCs accompanied by a malignant lesion. The patient background factors did not differ to a statistically extent according to age or sex. Cyst diameter in malignant cases tended to be larger in comparison to non-malignant cases (101.7 mm vs. 69.7 mm), and CT findings, such as mural nodule (66.6% vs. 33%) and wall calcification (66.7% vs. 27.3%), were more frequently reported in malignant cases. From these results, the evaluation of preoperative imaging findings, such as cyst diameter, mural nodules and calcification seems important for diagnosing potential malignancy.

**Table 1:** Clinical features of retroperitoneal isolated enteric duplication cyst in adults (*n* = 18)

Age (years)	49.9 ± 17.7 (19–75)
Sex (male:female)	6:12
Asymptomatic, yes	5 (28%)
Diameter of cyst (mm)	76.6 ± 35.2 (35–148)
Location (right:left)	5:11 Not described in 2 cases
Mural nodule, present	7 (44%)
Calcification, present	7 (41%) Not described in 1 case
Shape of cyst (unilocular:multilocular)	13:5
Malignancy, yes	6 (33%)
Operative method (laparotomy:laparoscopic)	11:7

Continuous date are shown as the mean ± standard deviation (range: minimum to maximum)

**Table 2:** Clinical features of malignant and non-malignant cases of retroperitoneal isolated enteric duplication cyst

	Malignant cases (*n* = 6)	Non-malignant cases (*n* = 12)
Age (average, years)	46.7 ± 15.9 (26–64)	36.6 ± 18.2 (19–75)
Sex (male:female)	2:4	4:8
Asymptomatic, yes	1 (17%)	4 (33%)
Diameter of cyst (average, mm)	101.7 ± 45.5	69.7 ± 31.0
Mural nodule, present	4 (67%)	4 (33%)
Calcification, present	4 (67%)	3 (27%)
Shape of cyst (unilocular:multilocular)	5:1	8:4

Continuous date are shown as the mean ± standard deviation (range: minimum to maximum)

As described above, our case is noteworthy as we were able to observe the imaging changes of the lesion over time over a relatively long period. There were two characteristic changes. The first change was the decreasing CT value inside the cystic lesion. The second was the pattern of growth that occurred over time. Radiologically, intracystic hemorrhage can be distinguished from a high CT value of ≥ 100 HU. Fukuhara et al. reported a duodenal EDC complicated with intracystic hemorrhage. They reported that the presence of ectopic gastric mucosa could cause the development of complications, such as bleeding and peptide ulcer [28]. Although Ildstad et al. noted in a pediatric case series that 35% of EDCs contained ectopic mucosa and 10% presented with gastrointestinal hemorrhage due to ectopic lesion [29], it could not be pathologically identified on the resected specimen in our case. This disease involves congenital malformations that are often found in the neonatal or prenatal period. On the other hand, the mechanism of cases that develop only in adulthood is unknown. Concerning the process in the present case, if the congenital EDCs continue to increase in response some acquired reasons, it is possible that intracystic bleeding may act as a trigger. However, the scientific evidence to support the above hypothesis cannot be established in this case, so it remains a matter for speculation.

There are various differential diagnoses of solitary cystic lesions in the retroperitoneal cavity. Among them, non-epithelial tumors are the most common; these include cystic lymphangioma, bronchial cyst, cystic teratoma and cystic mesothelioma. Epithelial tumors include epidermoid cyst, mucinous cystadenoma and cystadenocarcinoma [30]. Imaging evaluations are mainly used to predict the malignant potential of these lesions before surgery because of the difficulty in obtaining tissue. It is noteworthy that in all of the retroperitoneal cystic disease listed above, some malignant cases have been reported. In particular, in the case of mucinous cystadenoma or mucinous cystadenocarcinoma, a ruptured cyst could cause pseudomyxoma or cancerous peritonitis, which can be difficult to treat. Thus, when performing surgical treatment for retroperitoneal lesions, a careful and protective operation is required to remove the cyst without breaking the cyst wall, irrespective of the preoperative diagnosis. In this case, we performed laparoscopic surgery. The good field of view and magnifying effect with the procedure not only allowed us to avoid damaging the cyst wall, but were also useful for separating the cyst from adjacent structures and identifying the proper blood vessels of the duplication cyst.

## Conclusion

We reported a rare adult case of retroperitoneal isolated enteric duplication cyst, in which we could observe the changes of the image findings over time. It is difficult to obtain an accurate preoperative diagnosis of retroperitoneal cystic lesions. Therefore, we should consider the possibility of malignancy when determining the treatment strategy and perform a careful operation to avoid breaking the cyst wall, irrespective of the preoperative diagnosis.

## Data Availability

All data generated or analyzed during this study are included in this published article.

## Abbreviations

EDC: Enteric duplication cyst
IEDC: Isolated enteric duplication cyst
CA19-9: Carbohydrate antigen 19-9
CT: Computed tomography
HU: Hounsfield unit
